# Ameliorated Chameleon Algorithm-Based Shape Optimization of Disk Wang–Ball Curves

**DOI:** 10.3390/biomimetics10010003

**Published:** 2024-12-24

**Authors:** Yan Liang, Rui Yang, Xianzhi Hu, Gang Hu

**Affiliations:** 1 School of Technology, Xi’an Siyuan University, Xi’an 710038, China; 2 Xi’an Mingde Institute of Technology, College of General Education, Xi’an 710600, China; yangrui_rrr@163.com; 3 Division of Informationize Management, Xi’an University of Technology, Xi’an 710048, China; huxianzhi@xaut.edu.cn; 4 School of Computer Science and Engineering, Xi’an University of Technology, Xi’an 710048, China; hugang@xaut.edu.cn

**Keywords:** combined disk Wang–Ball curves, geometric continuity, shape optimization, energy minimization, ameliorated chameleon algorithm

## Abstract

The shape design and optimization of complex disk curves is a crucial and intractable technique in computer-aided design and manufacturing (CAD/CAM). Based on disk Wang–Ball (DWB) curves, this paper defines a novel combined disk Wang–Ball (CDWB) curve with constrained parameters and investigates the shape optimization of CDWB curves by using the multi-strategy ameliorated chameleon swarm algorithm (MCSA). Firstly, in order to meet the various shape design requirements, the CDWB curves consisting of *n* DWB curves are defined, and the G^1^ and G^2^ geometric continuity conditions for the curves are derived. Secondly, the shape optimization of CDWB curves is considered as a minimization problem with curve energy as the objective, and an optimization model is developed under the constraints of the splicing conditions. Finally, the meta-heuristic algorithm MCSA is introduced to solve the established optimization model to obtain the minimum energy value, and its performance is verified by comparison with other algorithms. The results of representative numerical examples confirm the effectiveness and competitiveness of the MCSA for the CDWB curve shape optimization problems.

## 1. Introduction

The cubic Ball basis function was first established for the CONSURF fuselage surface modeling tool [1,2,3]. Later on, Wang [4] and Said [5,6,7], respectively, promoted higher-order forms of the Ball basis functions. With an additional study by Hu et al., these two types of generalized Ball curves were, respectively, called Wang–Ball (WB) curves and Said–Ball (SB) curves [8]. At the same time, the paper points out that Wang–Ball curves not only have properties that are analogous to Bézier curves such as stability, symmetry, endpoint interpolation, and geometric invariance, but also significantly outperform SB curves and Bézier curves with respect to the recursive valuation, on the one hand, and degree elevation and reduction techniques on the other.

### 1.1. Relevant Research Objectives

The modeling of curves is an important research component in CAGD and has significant applications across a wide range of fields such as CAD/CAM, aerospace, and artificial intelligence. Geometry-based modeling is currently the main method used in CAD/CAM; however, in a floating-point environment only approximate results are usually obtained. In order to ensure computational accuracy and algorithmic robustness, Sederberg et al. introduced the interval approach to CAGD and proposed the concept of interval Bézier curves [9]. Later on, concepts such as interval Ball curves and interval B-spline curves were introduced, and their boundary representations and degree reductions were also studied [10,11,12]. Although the interval control capstone contains error information, the rectangular interval will expand during the calculation, and it will not have rotational symmetry in a two-dimensional space [13]. To this end, Lin et al. suggested that disks be used instead of control vertices, and proposed disk Bézier (DB) curves [14]. Subsequently, Chen [15] investigated the reduced degree of DB curves, employing both linear programming and optimization methods; the isometric approximation of DB curves was researched by Chen et al. [16]; and Seah et al. [17] showed the use of disk B-spline curves in artistic strokes and 2D animation. Hu et al. [18] constructed disk Wang–Ball (DWB) curves and investigated their respective degree reduction problem.

Unlike conventional curves, a disk curve is represented by control vertices, with real numbers representing the radius. When the radius of the control disk takes different values, the curve will have different thicknesses. Given the flexible presentation of the disk curve and good properties regarding the WB curve, the DWB curve has certain advantages in shaping design issues. However, in complex styling designs, multiple curve splices can better satisfy the design requirements than single curves. Therefore, part of the aim of this paper consists of defining the combined disk Wang–Ball (CDWB) curves and deriving the geometric continuity conditions for their G^1^ and G^2^ smooth splices.

In the shape design of curves, the smoothness of the curve is an important criterion for judging the quality of the shape. To obtain a smoother curve, the energy of the curve is often used as the objective function to constrain the curve shape, which is called the energy method [19]. The energy method is a physics-based modeling idea that treats the curve as an elastic spline and the strain energy of the elastic spline as the energy function of the curve. Due to the good smoothing effect of the energy method, Juhász adjusted the curve shape dictated by the mixture of control points and mixing functions through the energy function [20], and Hu et al. constructed a combined cubic generalized Ball (CCG-Ball) curve/surface and studied its shape optimization using minimized energy as the evaluation criterion [21,22]. Based on this, this paper will use the energy of the CDWB curves as the objective function and the relative G^1^, G^2^ geometric continuity of the smooth curve splice as the constraint to build an optimization model and solve it so that the resulting curve has the best shape. In light of the fact that the objective function in the optimizing model is complex and inspired by the literature [21], the meta-heuristic algorithm (MA) will be adopted to solve the established model in this paper.

### 1.2. Literature Survey of the Meta-Heuristic Algorithm

As one of the more popular optimization methods in recent years, most MAs are inspired by evolutionary laws, physical rules, and the population behavior of social organisms. For example, the genetic algorithm (GA) based on Darwin’s laws of evolution [23], the Newton–Raphson-based optimizer (NRBO) inspired by Newton–Raphson’s approach [24], the black widow optimization (BWO) algorithm derived from the evolutionary process of spider populations [25], and Coyote and Badger Optimization (CBO), which is associated with the cooperative behaviors observed in honey badgers and coyotes [26]. Optimization problems with different levels of complexity and different types have different demands on the performance of optimization algorithms, and therefore more novel algorithms and variants are constantly being developed. The football team training algorithm (FTTA) opens up new options for solving optimization problems that stem from the training methods of football teams [27]. Hu et al. modified the marine predators algorithm and adapted it for optimizing approximate developable surfaces [28]. The poplar optimization algorithm (POA) simulates the sexual and asexual reproduction mechanisms of poplar trees and has shown competitive performance for image segmentation [29]. HGWODE was proposed as a hybrid algorithm based on differential evolutionary (DE) [30] algorithms and GWO and applied to UAV path planning [31]. Atom search optimization (ASO), which takes its inspiration from fundamental molecular dynamics, has been successfully performed for hydrogeologic parameter estimation [32]. Motivated by the reduced order of SB curve, an enhanced chimp optimization algorithm has been proposed called SOCSCHOA [33].

The chameleon swarm algorithm (CSA) [34] is one new MA that models the predatory behavior of chameleons. The performance demonstrated on the test functions and engineering applications of CSA illustrates its competitiveness, and the combination of the two search methods gives it a strong exploratory capability. And, its demonstrated performance on test functions and engineering applications illustrates its competitiveness. Currently, CSA has been introduced successfully for short-term wind speed prediction [35], 3D CNN networks for volume segmentation [36], optimal configuration design for stand-alone microgrid systems [37], and medical image fusion [38], as well as for problems. However, the over-reliance on optimal values in the optimization search process makes CSA suffer from the problem of lack of population diversity and that of premature convergence. In addition, like most MAs, being caught in a local optimum when faced with difficult optimization questions is a major drawback that affects its ability to find an optimum. For this reason, Hu et al. proposed an enhanced hybrid algorithm called CCECSA by mixing CSA with the crisscross optimization algorithm in finding the amount of degree reduction in DWB curves [18]; R. Mostafa et al. exploited the consumption operator to boost the global search capability of CSA and used it for feature selection [39]. A hybrid method CSMO in which CSA and mayfly optimization (MO) are built has also been proposed to face the problem of economic scheduling of CHP [40]. Also, as an improved version of CSA, MCSA [41] introduces several strategies to boost its population diversity and local exploration capabilities, like fractional-order (FO) calculus, sinusoidal adjustment, and a crossover-based comprehensive learning (CCL) strategy. Extensive experiments in the literature have demonstrated that MCSA exhibits highly advantageous performance compared to some of the advanced MAs and other improved versions of CSA.

### 1.3. Paper Contribution and Organization

**Figure 1:**
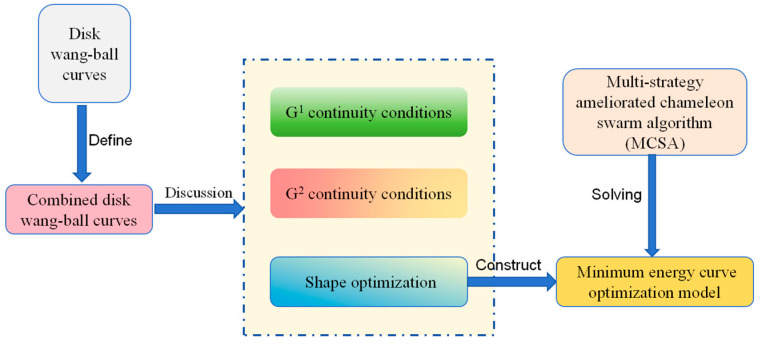
Main problems and methods.

This paper will employ MCSA as a means of solving the shape optimization problem of CDWB curves. The main problems and methods studied in this paper are shown in Figure 1. And chief contributions are summarized as follows:

1. The CDWB curve is defined on the basis of the concept of the DWB curve, together with a discussion of overall G^1^ and G^2^ continuity on the combined curve.
2. The shape optimization models of CDWB curves are established using the energy method. In order to seek a better optimization effect, the model is solved by introducing MCSA.
3. Three numerical examples are designed using CDWB curves, through which the optimization capability of MCSA is demonstrated.

The remaining sections of the arrangement are listed below. Section 2 introduces the definitions of DWB and CDWB curves, and discusses the geometric continuity conditions for CDWB curves. Section 3 first establishes the shape optimization models for CDWB curves, followed by a detailed introduction of MCSA, and finally proposes a method for solving CDWB curve shape optimization based on the MCSA and gives three numerical examples. The conclusion is presented in Section 4.

## 2. CDWB Curves

### 2.1. DWB Curves

**Definition** **1.** *In R^2^, given n + 1 control disks* $\left(P_{i})=(p_{i},r_{i}),(i=0,1,\cdots ,n\right)$*, the DWB curve with degree n is specifically given by*

(1) $$(W)(t)=\sum_{i=0}^{n}W_{i,n}(t)(P_{i})=\sum_{i=0}^{n}W_{i,n}(t)(p_{i},r_{i}),(0\leq t\leq 1),$$

where $p_{i}=(x_{i},y_{i})$*represents the control vertexes and r_i_ is the control radius, and*${\left\{W_{i,n}(t)\right\}}_{i=0}^{n}$*indicates the Wang–Ball basis functions, in which*

(2) $$W_{i,n}(t)=\left\{\begin{array}{l} {\left(2t\right)}^{i}{\left(1-t\right)}^{i+2},\;\;\;0\leq i\leq \left\lfloor n/2\right\rfloor -1\;,\\ {\left(2t\right)}^{\left\lfloor n/2\right\rfloor }{\left(1-t\right)}^{\left\lceil n/2\right\rceil },\;\;i=\left\lfloor n/2\right\rfloor \;,\\ {\left(2(1-t)\right)}^{\left\lfloor n/2\right\rfloor }t^{\left\lceil n/2\right\rceil },\;\;i=\left\lceil n/2\right\rceil \;,\\ W_{n-i}^{n}(1-t),\;\;\;\;\;\left\lceil n/2\right\rceil +1\leq i\leq n\;, \end{array}\right.$$

*where*$\left\lfloor x\right\rfloor$ and $\left\lceil x\right\rceil$*, respectively, refer to maximum integers less than or equal to *x* and a minimum integer greater than or equal to x.*

*Moreover, the form of Equation (1) can be formulated as follows:*

(3) $$(W)(t)=(C(t),R(t))=(\sum_{i=0}^{n}W_{i}^{n}(t)p_{i},\sum_{i=0}^{n}W_{i}^{n}(t)r_{i}),(0\leq t\leq 1),$$

*where **C**(t) and R(t), respectively, imply the center curve and radius function.*

**Theorem** **1.** *The DWB curve defined by Equation (1) shall satisfy the following endpoint properties:*

(4) $$\left\{\begin{array}{l} (W)(0)=(p_{0},r_{0}),\\ (W)(1)=(p_{n},r_{n}),\\ (W{)}^{\prime }(0)=2((p_{1},r_{1})-(p_{0},r_{0})),\\ (W{)}^{\prime }(1)=2((p_{n},r_{n})-(p_{n-1},r_{n-1})),\\ (W{)}^{\prime \prime }(0)=2((p_{0},r_{0})-6(p_{1},r_{1})+4(p_{2},r_{2})+(p_{n},r_{n}))(n\geq 4),\\ (W{)}^{\prime \prime }(1)=2((p_{0},r_{0})+4(p_{n-2},r_{n-2})-6(p_{n-1},r_{n-1})+(p_{n},r_{n}))(n\geq 4). \end{array}\right.$$

**Proof.**  A simple calculation of the Wang–Ball basis function with order n given in Equation (2) results in

$$W_{i,n}(0)=\left\{\begin{array}{l} 1,(i=0)\\ 0,(i\neq 0) \end{array}\right.,W_{i,n}(1)=\left\{\begin{array}{l} 1,(i=n)\\ 0,(i\neq n) \end{array}\right.,\;W_{i,n}^{\prime }(0)=\left\{\begin{array}{l} -2,(i=0)\\ 2,\;(i=1)\\ 0,\;(i=2,\cdots ,n) \end{array}\right.,W_{i,n}^{\prime }(1)=\left\{\begin{array}{l} 0,\;(i=0,\cdots ,n-2)\\ -2,(i=n-1)\\ 2,\;(i=n) \end{array}\right.,\;W_{i,n}^{\prime \prime }(0)=\left\{\begin{array}{l} 2,\;\;\;(i=0)\\ -12,\;(i=1)\\ 8,\;\;\;(i=2)\\ 0,\;\;\;(i=3,\cdots ,n-1)\\ 2,\;\;\;(i=n) \end{array}\right.,(n\geq 4),W_{i,n}^{\prime \prime }(1)=\left\{\begin{array}{l} 2,\;\;\;(i=0)\\ 0,\;\;\;(i=1,\cdots ,n-3)\\ 8,\;\;\;(i=n-2)\\ -12,\;(i=n-1)\\ 2,\;\;\;(i=n) \end{array}\right.,(n\geq 4).$$

With the combined Equation (1), the conclusion in Equation (4) can be deduced, and thus Theorem 1 is proved. □

### 2.2. Construction of CDWB Curves

Complex designs are often difficult to achieve with a single DWB curve, and the splicing of multiple curves can meet more of the design requirements. This section, therefore, defines the CDWB curve generated by stitching multiple DWB curves and discusses the stitching conditions.

**Definition** **2.** *Provided the following m + 1 nodes,*

$$\tau_{0}<\tau_{1}<\tau_{2}<\cdots <\tau_{j}<\tau_{j+1}<\cdots <\tau_{m-1}<\tau_{m},$$

*then the CDWB curve is expressed as*

(5) $$(\bar{W})(\tau )=\left\{\begin{array}{l} {\left(W\right)}_{1}(\frac{\tau -\tau_{0}}{h_{1}}),\;\;\;\tau \in [\tau_{0},\tau_{1}],\\ \cdots \cdots \\ {\left(W\right)}_{j}(\frac{\tau -\tau_{j-1}}{h_{j}}),\;\;\tau \in [\tau_{j-1},\tau_{j}]\\ \cdots \cdots \\ {\left(W\right)}_{m}(\frac{\tau -\tau_{m-1}}{h_{m}}),\;\;\tau \in [\tau_{m-1},\tau_{m}], \end{array}\right.,$$

*where* $h_{j}=\tau_{j}-\tau_{j-1}$.

The CDWB curve in Equation (5) as well can be abbreviated as

(6) $$\tilde{\Pi }:{\left(W\right)}_{j}(\frac{\tau -\tau_{j-1}}{h_{j}})=\sum_{i=0}^{n}\left(P_{i,j})W_{i,n}(\frac{\tau -\tau_{j-1}}{h_{j}}\right),(i=0,\cdots ,n,j=1,\cdots ,m)$$

where $\left(P_{i,j}\right)$ represents the *i* + 1th control disk of the *j*th DWB curve segment.

According to the representation of the DWB curve in Equation (3), the CDWB could also be shown as

(7) $$(\bar{W})(\tau )=(\bar{C}(\tau ),\bar{R}(\tau )),$$

where

(8) $$\bar{C}(\tau )=\left\{\begin{array}{l} C_{1}(\frac{\tau -\tau_{0}}{h_{1}}),\;\;\tau \in \left[\tau_{0},\tau_{1}\right],\\ \cdots \cdots \\ C_{j}(\frac{\tau -\tau_{j-1}}{h_{j}}),\;\;\tau \in \left[\tau_{j-1},\tau_{j}\right],\\ \cdots \cdots \\ C_{m}(\frac{\tau -\tau_{m-1}}{h_{m}}),\;\;\tau \in \left[\tau_{m-1},\tau_{m}\right], \end{array}\right.$$

(9) $$\bar{R}(\tau )=\left\{\begin{array}{l} R_{1}(\frac{\tau -\tau_{0}}{h_{1}}),\;\;\tau \in \left[\tau_{0},\tau_{1}\right],\\ \cdots \cdots \\ R_{j}(\frac{\tau -\tau_{j-1}}{h_{j}}),\;\;\tau \in \left[\tau_{j-1},\tau_{j}\right],\\ \cdots \cdots \\ R_{m}(\frac{\tau -\tau_{m-1}}{h_{m}}),\;\;\tau \in \left[\tau_{m-1},\tau_{m}\right]. \end{array}\right.$$

#### 2.2.1. CDWB Curves with G^1^ Geometric Continuity

**Theorem** **2.** *For a CDWB curve consisting of a combination of m DWB curves, when the control disks of the jth and j + 1th (j = 1, 2, ..., m − 1) curves satisfy the following constraint:*

(10) $$\left\{\begin{array}{l} (p_{0,j+1,},r_{0,j+1})=(p_{n,j,},r_{n,j}),\\ (p_{1,j+1},r_{1,j+1})=(\frac{h_{j+1}}{\alpha_{1}h_{j}}(p_{n,j}-p_{n-1,j})+p_{0,j+1},\frac{h_{j+1}}{\alpha_{2}h_{j}}(r_{n,j}-r_{n-1,j})+r_{0,j+1}), \end{array}\right.$$

*then the CDWB curve achieves G^1^ smooth continuity at the nodes* $\tau_{j}$, *where* $\alpha_{1}$ *and* $\alpha_{2}$ *are arbitrary constants, and*$\alpha_{1}>0,\alpha_{2}>0$*. If the CDWB curve reaches G^1^ smooth continuity at any node, then the CDWB curve is G^1^ smoothly continuous.*

**Proof.**  To make the *j*th and *j* + 1th DWB curves of $\left(\bar{W})(\tau )=(\bar{C}(\tau ),\bar{R}(\tau )\right)$ achieve G^1^ smooth continuity at the node $\tau_{j}$, they should first be guaranteed to be G^0^ continuous. This means that the last control disk of the *j*th DWB curve is equal to the first one of the *j* + 1th DWB curve, as follows:

(11) $$(p_{n,j},r_{n,j})=(p_{0,j+1},r_{0,j+1}).$$

Secondly, the G^1^ continuity should be satisfied at the junction $\tau_{j}$ of the CDWB curve, meaning that the *j*th and *j* + 1th curves should have a common tangent vector at $\tau_{j}$,

(12) $$({\bar{C}}^{\prime }(\tau_{j}^{-}),{\bar{R}}^{\prime }(\tau_{j}^{-}))=(\alpha_{1}{\bar{C}}^{\prime }(\tau_{j}^{+}),\alpha_{2}{\bar{R}}^{\prime }(\tau_{j}^{+})),$$

where $\alpha_{1}$ and $\alpha_{2}$ are arbitrary constants and $\alpha_{1}>0,\alpha_{2}>0$.

By the endpoint property of the DWB curve, it follows that

(13) $$\left\{\begin{array}{l} ({\bar{C}}^{\prime }(\tau_{j}^{-}),{\bar{R}}^{\prime }(\tau_{j}^{-}))=(\frac{2}{h_{j}}(p_{n,j}-p_{n-1,j}),\frac{2}{h_{j}}(r_{n,j}-r_{n-1,j})),\\ ({\bar{C}}^{\prime }(\tau_{j}^{+}),{\bar{R}}^{\prime }(\tau_{j}^{+}))=(\frac{2}{h_{j+1}}(p_{1,j+1}-p_{0,j+1}),\frac{2}{h_{j+1}}(r_{1,j+1}-r_{0,j+1})). \end{array}\right.$$

Integrating Equation (13) and Equation (12) results in

(14) $$(p_{1,j+1},r_{1,j+1})=(\frac{h_{j+1}}{\alpha_{1}h_{j}}(p_{n,j}-p_{n-1,j})+p_{0,j+1},\frac{h_{j+1}}{\alpha_{2}h_{j}}(r_{n,j}-r_{n-1,j})+r_{0,j+1}).$$

Thus, when the CDWB curve satisfies both Equations (11) and (14) at the node $\tau_{j}$, it is possible to make the splicing of the *j*th and the *j* + 1th curves achieve G^1^ smooth continuity, and Theorem 2 is proved. □

**Note** **1.** *In Theorem 1, the CDWB curve reaches the G^1^ smooth continuity condition in Equation (10), which is equivalent to*

(15) $$\left\{\begin{array}{l} p_{0,j+1}=p_{n,j},\\ p_{1,j+1}=\frac{h_{j+1}}{\alpha_{1}h_{j}}(p_{n,j}-p_{n-1,j})+p_{0,j+1}, \end{array}\right.$$

(16) $$\left\{\begin{array}{l} r_{0,j+1}=r_{n,j},\\ r_{1,j+1}=\frac{h_{j+1}}{\alpha_{2}h_{j}}(r_{n,j}-r_{n-1,j})+r_{0,j+1}. \end{array}\right.$$

**Figure 2:**
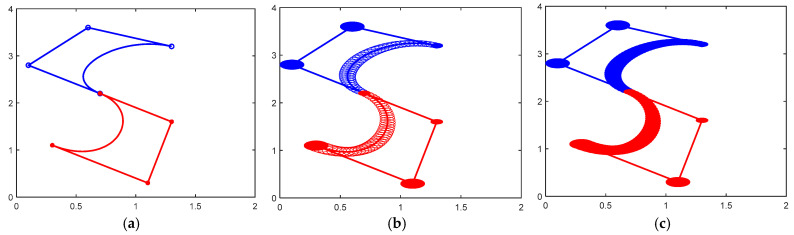
CDWB curve with overall G^1^ continuity ($\alpha_{1}=\alpha_{2}=1$). (**a**) The center curve; (**b**) the control disks; (**c**) the CDWB curve.

**Figure 3:**
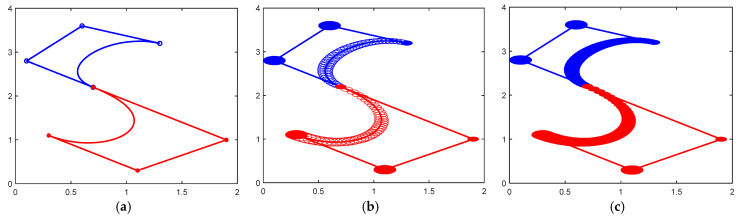
CDWB curve with overall G^1^ continuity ($\alpha_{1}=0.5,\alpha_{2}=0.6$). (**a**) The center curve; (**b**) the control disks; (**c**) the CDWB curve.

Figure 2 and Figure 3 show the design of the letter ‘S’ based on a CDWB curve, which is a combination of two DWB curves and satisfies the G^1^ smooth continuum as a whole. The difference between the two figures is in the values of the parameters $\alpha_{1}$ and $\alpha_{2}$. As can be observed from the graphs, there is also a significant difference in the effect produced when $\alpha_{1}$ and $\alpha_{2}$ are set to different values.

#### 2.2.2. CDWB Curves with G^2^ Geometric Continuity

**Theorem** **3.** *If the control disks of the jth and j + 1th adjacent curves (j = 1, 2, ..., m − 1) of the CDWB curve satisfies the following constraint:*

(17) $$\left\{\begin{array}{l} (p_{0,j+1},r_{0,j+1})=(p_{n,j},r_{n,j}),\\ (p_{1,j+1},r_{1,j+1})=(\frac{h_{j+1}}{\alpha_{1}h_{j}}(p_{n,j}-p_{n-1,j})+p_{0,j+1},\frac{h_{j+1}}{\alpha_{2}h_{j}}(r_{n,j}-r_{n-1,j})+r_{0,j+1}),\\ (p_{2,j+1},r_{2,j+1})=\\ \;(\frac{h_{j+1}^{2}}{4\alpha_{1}^{2}h_{j}^{2}}(p_{0,j}+4p_{n-2,j}-6p_{n-1,j}+p_{n,j})-\frac{1}{4}(p_{0,j+1}-6p_{1,j+1}+p_{n,j+1})-\frac{\beta_{1}h_{j+1}}{4\alpha_{1}^{2}}(p_{1,j+1}-p_{0,j+1})\\ \;\;,\frac{h_{j+1}^{2}}{4\alpha_{2}^{2}h_{j}^{2}}(r_{0,j}+4r_{n-2,j}-6r_{n-1,j}+r_{n,j})-\frac{1}{4}(r_{0,j+1}-6r_{1,j+1}+r_{n,j+1})-\frac{\beta_{2}h_{j+1}}{4\alpha_{2}^{2}}(r_{1,j+1}-r_{0,j+1})), \end{array}\right.$$

*then the CDWB curve is G^2^ continuity at knot* $\tau_{j}$, *where* $\alpha_{1},\alpha_{2},\beta_{1},\beta_{2}$ *are arbitrary constants and* $\alpha_{1}>0,\alpha_{2}>0$. *If the CDWB curve reaches G^2^ continuity at any node* $\tau_{j}(j=1,2,\cdots ,m)$*, it will be G^2^ continuity for the CDWB curve as a whole*.

**Proof.**  For CDWB curves, they should first satisfy G^1^ smooth continuity at the nodes if they are to achieve G^2^ continuity, i.e., Equation (10).

Second, respectively, denote by ***D***_1_ and ***D***_2_ as the binormal vectors of the *j*th and the *j* + 1th DWB curves; then, it follows that

(18) $$\left\{\begin{array}{l} D_{1}=({\bar{C}}^{\prime }(\tau_{j}^{-}),{\bar{R}}^{\prime }(\tau_{j}^{-}))\times ({\bar{C}}^{\prime \prime }(\tau_{j}^{-}),{\bar{R}}^{\prime \prime }(\tau_{j}^{-})),\\ D_{2}=({\bar{C}}^{\prime }(\tau_{j}^{+}),{\bar{R}}^{\prime }(\tau_{j}^{+}))\times ({\bar{C}}^{\prime \prime }(\tau_{j}^{+}),{\bar{R}}^{\prime \prime }(\tau_{j}^{+})). \end{array}\right.$$

G^2^ continuity further requires that ***D***_1_ and ***D***_2_ have the same orientation at the node $\tau_{j}$. According to Equations (12) and (18), it follows that the four vectors $\left({\bar{C}}^{\prime }(\tau_{j}^{-}),{\bar{R}}^{\prime }(\tau_{j}^{-})\right)$, $\left({\bar{C}}^{\prime \prime }(\tau_{j}^{-}),{\bar{R}}^{\prime \prime }(\tau_{j}^{-})\right)$, $\left({\bar{C}}^{\prime }(\tau_{j}^{+}),{\bar{R}}^{\prime }(\tau_{j}^{+})\right)$, $\left({\bar{C}}^{\prime \prime }(\tau_{j}^{+}),{\bar{R}}^{\prime \prime }(\tau_{j}^{+})\right)$ are coplanar. Thus,

(19) $$({\bar{C}}^{\prime \prime }(\tau_{j}^{-}),{\bar{R}}^{\prime \prime }(\tau_{j}^{-}))=((u_{1}{\bar{C}}^{\prime \prime }(\tau_{j}^{+}),u_{2}{\bar{R}}^{\prime \prime }(\tau_{j}^{+}))+(\beta_{1}{\bar{C}}^{\prime }(\tau_{j}^{+}),\beta_{2}{\bar{R}}^{\prime }(\tau_{j}^{+})).$$

And, alternatively,

(20) $$({\bar{C}}^{\prime \prime }(\tau_{j}^{-}),{\bar{R}}^{\prime \prime }(\tau_{j}^{-}))=(u_{1}{\bar{C}}^{\prime \prime }(\tau_{j}^{+})+\beta_{1}{\bar{C}}^{\prime }(\tau_{j}^{+}),u_{2}{\bar{R}}^{\prime \prime }(\tau_{j}^{+})+\beta_{2}{\bar{R}}^{\prime }(\tau_{j}^{+})),$$

where *u*_1_, *u*_2_, *β*_1_, *β*_2_ are arbitrary constants and *u*_1_ > 0, *u*_2_ > 0.

At node $l_{j}$, supposing that the curvatures of the *j*th and the *j* + 1th center curves are $\kappa c(\tau_{j}^{-})$ and $\kappa c(\tau_{j}^{+})$, respectively, then

(21) $$\left\{\begin{array}{l} \kappa c(\tau_{j}^{-})=\frac{\left|{\bar{C}}^{\prime }(\tau_{j}^{-})\times {\bar{C}}^{\prime \prime }(\tau_{j}^{-})\right|}{{\left|{\bar{C}}^{\prime }(\tau_{j}^{-})\right|}^{3}},\\ \kappa c(\tau_{j}^{+})=\frac{\left|{\bar{C}}^{\prime }(\tau_{j}^{+})\times {\bar{C}}^{\prime \prime }(\tau_{j}^{+})\right|}{{\left|{\bar{C}}^{\prime }(\tau_{j}^{+})\right|}^{3}}. \end{array}\right.$$

In accordance with Equations (12) and (20), the curvature $\kappa c(\tau_{j}^{-})$ is given as

(22) $$\begin{array}{l} \kappa c(\tau_{j}^{-})=\frac{\left|\alpha_{1}{\bar{C}}^{\prime }(\tau_{j}^{+})\times [u_{1}{\bar{C}}^{\prime \prime }(\tau_{j}^{+})+\beta_{1}{\bar{C}}^{\prime }(\tau_{j}^{+})]\right|}{{\alpha_{1}}^{3}{\left|{\bar{C}}^{\prime }(\tau_{j}^{+})\right|}^{3}}\\ \;\;\;\;=\frac{u_{1}\left|{\bar{C}}^{\prime }(\tau_{j}^{+})\times {\bar{C}}^{\prime \prime }(\tau_{j}^{+})\right|}{{\alpha_{1}}^{2}{\left|{\bar{C}}^{\prime }(\tau_{j}^{+})\right|}^{3}}. \end{array}$$

When the *j*th curve is G^2^ continuous with the *j* + 1th curve at the splice point, $\kappa c(\tau_{j}^{-})$ and $\kappa c(\tau_{j}^{+})$ carries an identical value. So, it follows that $u_{1}=\alpha_{1}^{2}$, according to Equations (21) and (22).

For the radius function, the same is true for $u_{2}=\alpha_{2}^{2}$. Substituting them into Equation (20) gives

(23) $$({\bar{C}}^{\prime \prime }(\tau_{j}^{-}),{\bar{R}}^{\prime \prime }(\tau_{j}^{-}))=(\alpha_{1}^{2}{\bar{C}}^{\prime \prime }(\tau_{j}^{+})+\beta_{1}{\bar{C}}^{\prime }(\tau_{j}^{+}),\alpha_{2}^{2}{\bar{R}}^{\prime \prime }(\tau_{j}^{+})+\beta_{2}{\bar{R}}^{\prime }(\tau_{j}^{+})).$$

Finally, by the endpoint property of the CDWB curve, there is

(24) $$\left\{\begin{array}{l} ({\bar{C}}^{\prime \prime }(\tau_{j}^{-}),{\bar{R}}^{\prime \prime }(\tau_{j}^{-}))=(\frac{2}{h_{j}^{2}}(p_{0,j}+4p_{n-2,j}-6p_{n-1,j}+p_{n,j}),\frac{2}{h_{j}^{2}}(r_{0,j}+4r_{n-2,j}-6r_{n-1,j}+r_{n,j})),\\ ({\bar{C}}^{\prime \prime }(\tau_{j}^{+}),{\bar{R}}^{\prime \prime }(\tau_{j}^{+}))=(\frac{2}{h_{j+1}^{2}}(p_{0,j+1}-6p_{1,j+1}+4p_{2,j+1}+p_{n,j+1}),\frac{2}{h_{j+1}^{2}}(r_{0,j+1}-6r_{1,j+1}+4r_{2,j+1}+r_{n,j+1})). \end{array}\right.$$

The result of Equation (13) is combined and substituted into Equation (24) to obtain

(25) $$\begin{array}{l} (p_{2,j+1},r_{2,j+1})=\\ \;\;(\frac{h_{j+1}^{2}}{4\alpha_{1}^{2}h_{j}^{2}}(p_{0,j}+4p_{n-2,j}-6p_{n-1,j}+p_{n,j})-\frac{1}{4}(p_{0,j+1}-6p_{1,j+1}+p_{n,j+1})-\frac{\beta_{1}h_{j+1}}{4\alpha_{1}^{2}}(p_{1,j+1}-p_{0,j+1})\\ \;\;,\frac{h_{j+1}^{2}}{4\alpha_{2}^{2}h_{j}^{2}}(r_{0,j}+4r_{n-2,j}-6r_{n-1,j}+r_{n,j})-\frac{1}{4}(r_{0,j+1}-6r_{1,j+1}+r_{n,j+1})-\frac{\beta_{2}h_{j+1}}{4\alpha_{2}^{2}}(r_{1,j+1}-r_{0,j+1})), \end{array}$$

where $\alpha_{1},\alpha_{2},\beta_{1},\beta_{2}$ are arbitrary constants and $\alpha_{1}>0,\alpha_{2}>0$.

Thus, when the CDWB curve satisfies both Equations (10) and (25) at the node $\tau_{j}$, it is possible to make the splicing of the *j*th DWB curve with the *j* + 1th DWB curve achieve G^2^ smooth continuity, and Theorem 3 is proved. □

**Note** **2.** *The G^2^ continuity condition in Theorem 2 is equivalent to*

(26) $$\left\{\begin{array}{l} p_{0,j+1}=p_{n,j},\\ p_{1,j+1}=\frac{h_{j+1}}{\alpha_{1}h_{j}}(p_{n,j}-p_{n-1,j})+p_{0,j+1},\\ p_{2,j+1}=\frac{h_{j+1}^{2}}{4\alpha_{1}^{2}h_{j}^{2}}(p_{0,j}+4p_{n-2,j}-6p_{n-1,j}+p_{n,j})-\frac{1}{4}(p_{0,j+1}-6p_{1,j+1}+p_{n,j+1})\\ \;\;\;-\frac{\beta_{1}h_{j+1}}{4\alpha_{1}^{2}}(p_{1,j+1}-p_{0,j+1}), \end{array}\right.$$

(27) $$\left\{\begin{array}{l} r_{0,j+1}=r_{n,j},\\ r_{1,j+1}=\frac{h_{j+1}}{\alpha_{2}h_{j}}(r_{n,j}-r_{n-1,j})+r_{0,j+1},\\ r_{2,j+1}=\frac{h_{j+1}^{2}}{4\alpha_{2}^{2}h_{j}^{2}}(r_{0,j}+4r_{n-2,j}-6r_{n-1,j}+r_{n,j})-\frac{1}{4}(r_{0,j+1}-6r_{1,j+1}+r_{n,j+1})\\ \;\;\;-\frac{\beta_{2}h_{j+1}}{4\alpha_{2}^{2}}(r_{1,j+1}-r_{0,j+1}). \end{array}\right.$$

**Figure 4:**
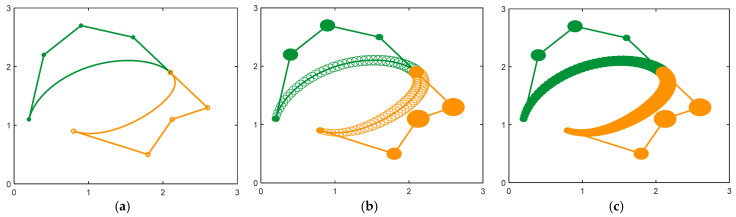
CDWB curve with overall G^2^ continuity ($\alpha_{1}=\alpha_{2}=\beta_{1}=1,\beta_{2}=8$). (**a**) The center curve; (**b**) the control disks; (**c**) the CDWB curve.

**Figure 5:**
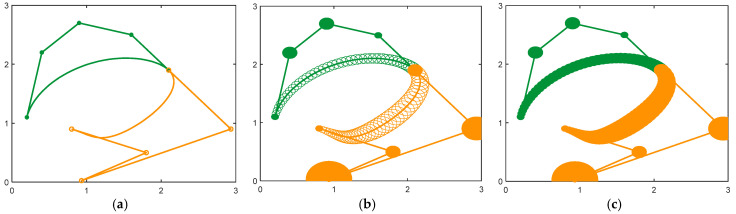
The CDWB curve with overall G^2^ continuity ($\alpha_{1}=0.6,\alpha_{2}=0.5,\beta_{1}=0.3,\beta_{2}=2$). (**a**) The center curve; (**b**) the control disks; (**c**) the CDWB curve.

Figure 4 and Figure 5 show the design “C” curve using a CDWB curve that satisfies the G^2^ continuum as a whole, and they show the profile shape for different parameter values.

**Figure 6:**
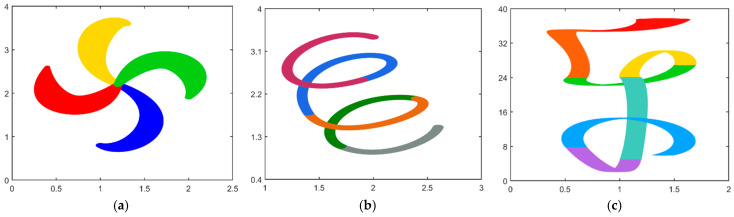
Modeling based on CDWB curves. (**a**) Windmill; (**b**) spring; (**c**) Chinese character “乐”.

By adjusting the control vertices and radii, multiple DWB curves combined to create CDWB curves that satisfy different continuity conditions, allowing various shapes to be designed, as shown in Figure 6.

## 3. Shape Optimization of CDWB Curves

### 3.1. Modeling of CDWB Curves Shape Optimization

Considering the curve as an elastic spline, the strain energy of the elastic spline is available as its energy function. Typically, curves with smaller energy values have better overall smoothness. As described in Section 2.1, a DWB curve consists of a center curve and radius function, and then its energy, as well, can take the form of the energy values of these two components. Therefore, the energy value $Energy_{CR}$ of a DWB curve is defined as follows:

(28) $$Energy_{CR}=Energy_{C}+Energy_{R},$$

where $Energy_{C}$ and $Energy_{R}$ denote the energy of C(t) and the energy of *R*(*t*), respectively, with the following mathematical equations:

(29) $$Energy_{C}=\int kc^{2}(s)ds=\int_{0}^{1}\frac{{\left(x^{\prime }(t)y^{\prime \prime }(t)-y^{\prime }(t)x^{\prime \prime }(t)\right)}^{2}}{{\left(x^{\prime }{\left(t\right)}^{2}+y^{\prime }{\left(t\right)}^{2}\right)}^{5/2}}dt,$$

where *kc* stands for the curvature of C(t),

(30) $$Energy_{R}=\int ||R^{\prime \prime }(t)|{|}^{2}dt=\int_{0}^{1}||(\sum_{i=0}^{n}W_{i,n}r_{i}{)}^{\prime \prime }|{|}^{2}dt.$$

To achieve the overall G^1^, G^2^ smooth splice of the CDWB curve, optimization models of $\left(\bar{W})(l)=(\bar{C}(l),\bar{R}(l)\right)$ based on the smoothest curve can be established. $\tilde{E}nergy_{CR}$ denotes the energy of the CDWB curve $\left(\bar{W})(l\right)$ as a whole, and the energies of $\bar{C}(l)$ and $\bar{R}(l)$ are indicated by $\tilde{E}nergy_{C}$ and $\tilde{E}nergy_{R}$, respectively; then the models will be as follows:

(a) Whole G^1^ smooth blending:

(31) $$\begin{array}{l} \mathrm{Minimize}\;\tilde{E}nergy_{CR}=\tilde{E}nergy_{C}+\tilde{E}nergy_{R}=\sum_{j=1}^{m}Energy_{C_{j}}+\sum_{j=1}^{m}Energy_{R_{j}}\\ \;\;\;\;\;=\sum_{j=1}^{m}\int_{\tau_{j-1}}^{\tau_{j}}\frac{{\left(x_{j}^{\prime }(\frac{\tau -\tau_{j-1}}{h_{j}})y_{j}^{\prime \prime }(\frac{\tau -\tau_{j-1}}{h_{j}})-y_{j}^{\prime }(\frac{\tau -\tau_{j-1}}{h_{j}})x_{j}^{\prime \prime }(\frac{\tau -\tau_{j-1}}{h_{j}})\right)}^{2}}{{\left(x_{j}^{\prime }{\left(\frac{\tau -\tau_{j-1}}{h_{j}}\right)}^{2}+y_{j}^{\prime }{\left(\frac{\tau -\tau_{j-1}}{h_{j}}\right)}^{2}\right)}^{5/2}}d\tau \\ \;\;\;\;\;+\sum_{j=1}^{m}\int_{\tau_{j-1}}^{\tau_{j}}||R_{j}^{\prime \prime }(\frac{\tau -\tau_{j-1}}{h_{j}})|{|}^{2}d\tau , \end{array}\;s.t.\;\left\{\begin{array}{l} (p_{0,j+1},r_{0,j+1})=(p_{n,j},r_{n,j}),\\ (p_{1,j+1},r_{1,j+1})=(\frac{h_{j+1}}{\alpha_{1}h_{j}}(p_{n,j}-p_{n-1,j})+p_{0,j+1},\frac{h_{j+1}}{\alpha_{2}h_{j}}(r_{n,j}-r_{n-1,j})+r_{0,j+1}), \end{array}\right.$$

where $\alpha_{1},\alpha_{2}$ are arbitrary constants and $\alpha_{1}>0$, $\alpha_{2}>0$.

(b) Whole G^2^ smooth blending:

(32) $$\begin{array}{c} \mathrm{Minimize}\;\tilde{E}nergy_{CR}=\sum_{j=1}^{m}\int_{\tau_{j-1}}^{\tau_{j}}\frac{{\left(x_{j}^{\prime }(\frac{\tau -\tau_{j-1}}{h_{j}})y_{j}^{\prime \prime }(\frac{\tau -\tau_{j-1}}{h_{j}})-y_{j}^{\prime }(\frac{\tau -\tau_{j-1}}{h_{j}})x_{j}^{\prime \prime }(\frac{\tau -\tau_{j-1}}{h_{j}})\right)}^{2}}{{\left(x_{j}^{\prime }{\left(\frac{\tau -\tau_{j-1}}{h_{j}}\right)}^{2}+y_{j}^{\prime }{\left(\frac{\tau -\tau_{j-1}}{h_{j}}\right)}^{2}\right)}^{5/2}}d\tau \\ +\sum_{j=1}^{m}\int_{\tau_{j-1}}^{\tau_{j}}||R_{j}^{\prime \prime }(\frac{\tau -\tau_{j-1}}{h_{j}})|{|}^{2}d\tau , \end{array}\;s.t.\;\left\{\begin{array}{l} (p_{0,j+1},r_{0,j+1})=(p_{n,j},r_{n,j}),\\ (p_{1,j+1},r_{1,j+1})=(\frac{h_{j+1}}{\alpha_{1}h_{j}}(p_{n,j}-p_{n-1,j})+p_{0,j+1},\frac{h_{j+1}}{\alpha_{2}h_{j}}(r_{n,j}-r_{n-1,j})+r_{0,j+1}),\\ \left(p_{2,j+1},r_{2,j+1})=(\frac{h_{j+1}^{2}}{4\alpha_{1}^{2}h_{j}^{2}}(p_{0,j}+4p_{n-2,j}-6p_{n-1,j}+p_{n,j})-\frac{1}{4}(p_{0,j+1}-6p_{1,j+1}+p_{n,j+1}\right)\\ \;\;\;\;\;\;-\frac{\beta_{1}h_{j+1}}{4\alpha_{1}^{2}}(p_{1,j+1}-p_{0,j+1})\;,\frac{h_{j+1}^{2}}{4\alpha_{2}^{2}h_{j}^{2}}(r_{0,j}+4r_{n-2,j}-6r_{n-1,j}+r_{n,j})\\ \;\;\;\;\;\;-\frac{1}{4}(r_{0,j+1}-6r_{1,j+1}+r_{n,j+1})-\frac{\beta_{2}h_{j+1}}{4\alpha_{2}^{2}}(r_{1,j+1}-r_{0,j+1})), \end{array}\right.$$

where $\alpha_{1},\alpha_{2},\beta_{1},\beta_{2}$ are arbitrary constants and $\alpha_{1}>0$, $\alpha_{2}>0$.

### 3.2. Mathematical Model of MCSA

MCSA is an improved algorithm based on CSA, which is suggested as an attempt to overcome the weaknesses of CSA, like becoming stuck in local optima, lack of population diversity, and premature convergence [41]. Three strategies are introduced in MCSA: the flexible adjustment of parameters by the sine function instead of its fixed value to balance the exploratory and exploitation better, FO calculus, using the historical iterations to accelerate global convergence, and the CCL strategy, mutating the current optimum of individuals to increase population diversity. *N* and *d* represent the population size and dimension, respectively. And $y_{i}^{g}=[y_{i,1}^{g},y_{i,2}^{g},\ldots ,y_{i,d}^{g}]$ indicates the position of chameleon *i.* Given a maximum number of iterations *Maxg*, MCSA iterates to find the optimal value based on the following model.

#### 3.2.1. Searching Stage Guided by Sinusoidal Adjustment

The search phase of the CSA simulates the behavior of chameleons wandering around deserts, woods, and so on looking for prey. It is updated by using optimal individual guidance or random wandering under the influence of perceived probability Pp. In this case, the impact of the global optimal on the current optimal of the individual depends on the value of the parameter *p*_1_. And the original paper experimentally determined a fixed value for it. It is well known that the values of the parameters taken in the face of different problems can have a significant impact.

As a periodic function, the sine function can also change the direction of exploration while adjusting the value of the parameter. The use of sinusoidal adjustment instead of a fixed value of the parameter *p*_1_ provides a degree of flexibility in the value of the parameter within the allowed range and makes full use of the search area. Thus, the combination of the two at this stage produces the following equation for the position update.

(33) $$y_{i}^{g+1}=\left\{\begin{array}{l} y_{i}^{g}+p_{1}(P_{i}^{g}-G^{g})r_{2}+p_{2}(G^{g}-y_{i}^{g})r_{1}\;r_{i}\geq Pp,\\ y_{i}^{g}+\mu ((ub-lb)r_{3}+lb)\mathrm{sgn}(rand-0.5)\;\;r_{i}<Pp, \end{array}\right.$$

where ***ub*** and ***lb*** denote the upper and lower bounds of the variables. $P_{i}^{g}$ denotes the best position of individual *i*, $G^{g}$ denotes the global best. $p_{2}$ is a constant. $r_{1}$, $r_{2}$, $r_{3}$ and $r_{i}$ are uniformly generated in the range [0, 1]. *p*_1_ and μ are calculated using Equations (34) and (35), respectively;

(34) $$p_{1}=\frac{1}{2}(\sin(2\pi \times freq\times t)\frac{g}{Maxg}+1),$$

where *freq* denotes the frequency,

(35) $$\mu =\gamma e^{(\frac{-bg}{Maxg})\beta },$$

in which γ, b, and β are constants.

#### 3.2.2. Rotation of Chameleon’s Eyes

With the ability to rotate independently, the two eyes of the chameleon possess a range of vision. It takes advantage of the oversized perspective to locate the prey and then rotate and move quickly to the prey’s location. The mathematical model for this phase is shown in Equation (36).

(36) $$y_{i}^{g+1}=yr_{i}^{g}+{\bar{y}}_{i}^{g},$$

where $yr_{i}^{t}$ stands for the coordinates of the rotation center, as shown in the following equation:

(37) $$yr_{i}^{g}=m\times yc_{i}^{g},$$

where $yc_{i}^{t}$ and m, respectively, represent the central coordinates and rotation matrix at the *g*th generation.

#### 3.2.3. Attacking Stage in Combination with FO Calculus

The chameleon’s tongue can extend twice as far as its own body and relies on the powerful suction produced by the tip of the tongue to suck in its prey. The speed of the chameleon is calculated by the following equation:

(38) $$v_{i}^{g+1}=\omega v_{i}^{g}+c_{1}(G^{g}-y_{i}^{g})r_{1}+c_{2}(P_{i}^{g}-y_{i}^{g})r_{2}$$

where ω is the inertia weight:

(39) $$\omega ={\left(1-\frac{g}{Maxg}\right)}^{\left(\rho \sqrt{\frac{g}{Maxg}}\right)},$$

where ρ refers to a positive value dominating the ability to exploit.

The combination of FO calculus and evolutionary methods is a way to make full use of the speed term generated by historical iterations to accelerate convergence. Thus, MCSA combines FO calculus with the velocity update formula in standard CSA to produce a hybrid form, as follows:

(40) $$\begin{array}{l} v_{i}^{g+1}=\alpha v_{i}^{g}+\frac{1}{2}\alpha (1-\alpha )v_{i}^{g-1}+\frac{1}{6}\alpha (1-\alpha )(2-\alpha )v_{i}^{g-2}\\ \;\;+\frac{1}{24}\alpha (1-\alpha )(2-\alpha )(3-\alpha )v_{i}^{g-3}+c_{1}(G^{g}-y_{i}^{g})r_{1}+c_{2}(P_{i}^{g}-y_{i}^{g})r_{2}, \end{array}$$

where the order α lies between [0,1].

As shown in Equation (40), the attack velocity of the chameleon is updated using the velocity term of historical iterations to accelerate the convergence trend. At the same time, it updates the position of the chameleon depending on the attack speed, which can be calculated using Equation (41):

(41) $$y_{i}^{g+1}=y_{i}^{g}+\left({\left(v_{i}^{g}\right)}^{2}-{\left(v_{i}^{g-1}\right)}^{2}\right)/\left(2a\right),$$

(42) $$a=2590\times (1-e^{-\log(g)}).$$

#### 3.2.4. CCL Strategy

During the iteration, the entire population’s search direction relies on the optimal individual to move forward. As the optimum is approached, the individual optimum, which lacks empirical information and the ability to learn independently, may become trapped in a local optimum. With this in mind, MCSA introduces a CCL strategy. As a variation strategy, the CCL strategy uses the information shared among different individuals and dimensions as a means of generating new individuals which can effectively improve the problem of dimensional stagnation. The mutation operation creates a new individual, $Np_{i}^{g}$, as shown in Equation (43),

(43) $$Np_{i}^{g}=\left\{\begin{array}{l} r_{1}p_{i}^{g}+(1-r_{1})p_{l}^{g}+c(p_{i}^{g}-p_{l}^{g}),\;\mathrm{if}\;rand\leq \lambda_{c},\\ r_{2}p_{i,j}^{g}+(1-r_{2})p_{i,j_{1}}^{g},\;\;\;\;\;\;\mathrm{otherwise}, \end{array}\right.$$

where *c* belongs to [−1,1] and *r_i_* (*i* = 1,2) is a random number between [0,1]. $j_{1}$ is a randomly chosen integer in [1,*d*] and $\lambda_{c}$ is cross-probability.

Subsequently, the greedy operation is executed and the better individual goes to the next iteration. That is,

(44) $$p_{i}^{g}=\left\{\begin{array}{l} Np_{i}^{g},\;\mathrm{if}\;(fit(Np_{i}^{g})<fit(p_{i}^{g})),\\ p_{i}^{g},\;\;\;\mathrm{else}. \end{array}\right.$$

A more detailed description of the MCSA is provided in Algorithm 1.**Algorithm 1:** Pseudo-code of MCSA Input: Related parameters, such as *d*, *N*, *Maxg*, *Pp*, α, $\lambda_{c}$, freqOutput: Optimal fitness value *fit_best_*1: Randomly initialize2: Calculate the fitness values of each chameleon’s position, record the best value 3: while (g<Maxg) do4:  Define the value of $p_{1}$, μ according to Equations (34) and (35)5:  Define the inertia weight ω and the acceleration rate a according to Equations (39) and (42)6:  for i=1:N do 7:    $y_{i}^{g+1}=\left\{\begin{array}{l} y_{i}^{g}+p_{1}(P_{i}^{g}-G^{g})r_{2}+p_{2}(G^{g}-y_{i}^{g})r_{1}\;r_{i}\geq Pp\\ y_{i}^{g}+\mu ((ub-lb)r_{3}+lb)\mathrm{sgn}(rand-0.5)\;\;r_{i}<Pp \end{array}\right.$8:    $y_{i}^{g+1}=yr_{i}^{g}+{\bar{y}}_{i}^{g}$9:    if (g≤4) do10:     $v_{i}^{g+1}=\omega v_{i}^{g}+c_{1}(G^{g}-y_{i}^{g})r_{1}+c_{2}(P_{i}^{g}-y_{i}^{g})r_{2}$11:    else12:     $\begin{array}{l} v_{i}^{g+1}=\alpha v_{i}^{g}+\frac{1}{2}\alpha (1-\alpha )v_{i}^{g-1}+\frac{1}{6}\alpha (1-\alpha )(2-\alpha )v_{i}^{g-2}\\ \;\;+\frac{1}{24}\alpha (1-\alpha )(2-\alpha )(3-\alpha )v_{i}^{g-3}+c_{1}(G^{g}-y_{i}^{g})r_{1}+c_{2}(P_{i}^{g}-y_{i}^{g})r_{2} \end{array}$13:    end if14:   $y_{i}^{g+1}=y_{i}^{g}+\left({\left(v_{i}^{g}\right)}^{2}-{\left(v_{i}^{g-1}\right)}^{2}\right)/\left(2a\right)$15:  end for16:  Compute the fitness values and update the best value17:  Find the best position $p_{i}^{g}$ for each individual so far18:  for i=1:N do19:   $Np_{i}^{g}=\left\{\begin{array}{l} r_{1}p_{i}^{g}+(1-r_{1})p_{l}^{g}+c(p_{i}^{g}-p_{l}^{g}),\;\mathrm{if}\;rand\leq \lambda_{c}\\ r_{2}p_{i,j}^{g}+(1-r_{2})p_{i,j_{1}}^{g},\;\;\;\;\;\;\mathrm{otherwise} \end{array}\right.$20:    if ($fit(Np_{i}^{g})<fit(p_{i}^{g})$) do21:     $p_{i}^{g}=Np_{i}^{g}$22:   end if23:  end for24:   g=g+125: end while


### 3.3. Steps for Solving the Optimization Models by MCSA

Using the minimized curve energy as the objective function, we will employ MCSA on the solution of the optimization model formulated in Section 3.1. The exact implementation steps take place along the following lines:

Step1: Set parameter values and generate the initial population with random initialization.

Step2: The energy function E of the CDWB curve is taken into account as the objective function; calculate the fitness value.

Step3: Calculate the values of $p_{1}$, μ on the basis of Equations (34) and (35), respectively, and identify the chameleon’s position using Equation (33).

Step4: Calculate the new location of each chameleon after the rotation from Equation (36).

Step5: Calculate the inertia weights ω according to Equation (40) and the acceleration according to Equation (43). If the number of iterations g≤4, use Equation (38) to determine the speed of attack; otherwise, apply Equation (40). Then, the positional transformation after the attack is calculated in accordance with Equation (41).

Step6: Calculate the fitness value, and determine $p_{i}^{g}$ and the optimal solution.

Step7: Execute the CCL strategy to generate a new individual optimum $Np_{i}^{g}$ using Equation (43). If $E(Np_{i}^{g})<E(p_{i}^{g})$, replace $Np_{i}^{g}$ with $p_{i}^{g}$ for the next iteration. Otherwise, leave it unchanged.

Step8: Determine whether the termination condition g<Maxg holds. If it holds, *g* = *g* + 1 while switching to step 3. Alternatively, output the optimal result at the end of the iteration.

**Figure 7:**
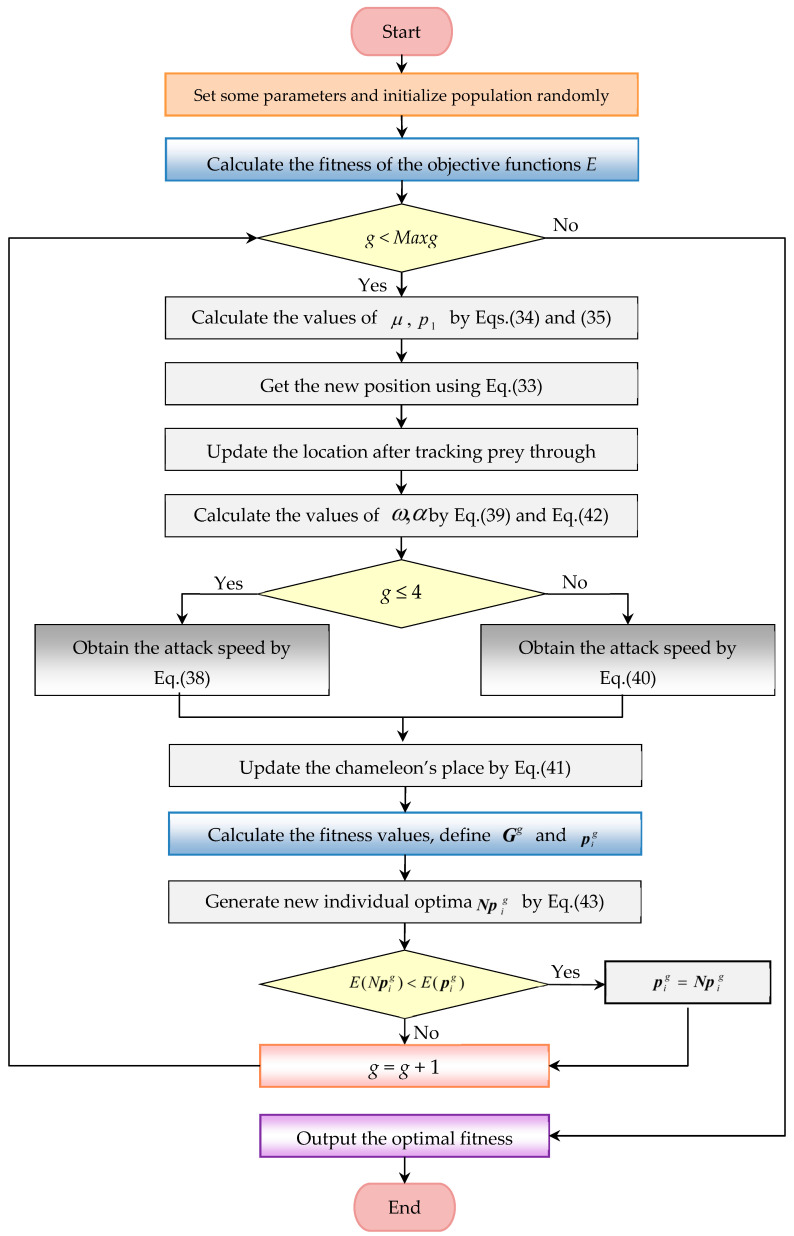
Flow chart for solving the energy minimum model based on MCSA.

A flow chart of MCSA used to solve the CDWB curves optimized model is given in Figure 7.

### 3.4. Numerical Examples

In this section, the optimization models developed in Section 3.1 are solved using MAs. The validity of MCSA is verified by 3 numerical examples with different successive conditions and levels of complexity. In addition, a number of advanced algorithms are selected for comparison, including the Sine Cosine Algorithm (SCA) [42], DE, White Shark Optimizer (WSO) [43], Arithmetic Optimization Algorithm (AOA) [44], Golden jackal optimization (GJO) [45], Sooty Tern Optimization Algorithm (STOA) [46], Greywolf optimizer (GWO) [47] and Multi-verse optimizer (MVO) [48]. The population size for each algorithm in the numerical experiments is 50.

**Example** **1.** *This is an example of optimizing the shape of a Chinese character “gong” designed from a CDWB curve with thickness to meet the overall G^1^ smooth continuity. This CDWB curve contains 4 DWB curves. To satisfy the overall G^1^ continuity, the first two control disks of curve (**W**)_j_ (j = 2,3,4) satisfy Equation (41) to ensure smooth continuity at the nodes. The curve (**W**)_1_ and the ith control disk of the jth (j = 2,3,4) curve are first given (i = 2,3,4), and then the corresponding energy minimization model is built according to Equation (31).*

**Figure 8:**
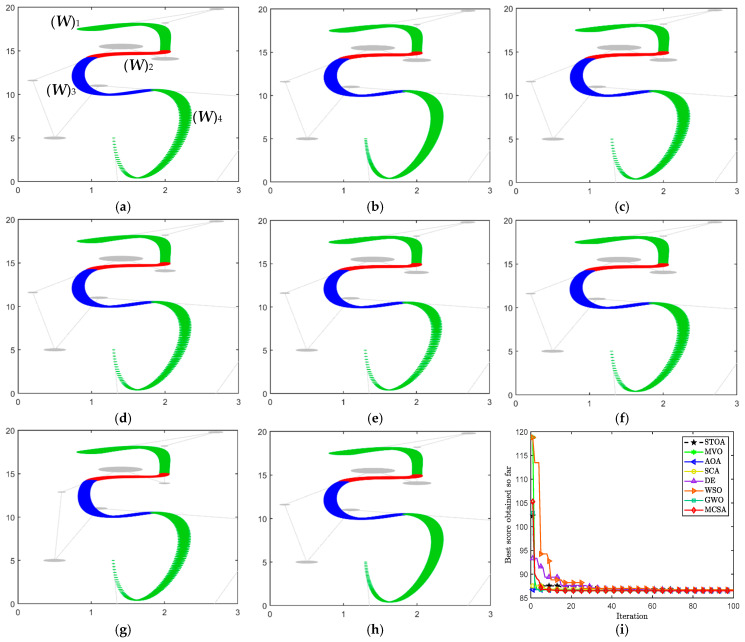
Chinese character “弓” based on CDWB curve with thickness. (**a**) GWO, (**b**) STOA, (**c**) MVO. (**d**) AOA, (**e**) SCA, (**f**) DE. (**g**) WSO, (**h**) MCSA, (**i**) convergence curves.

The optimization results obtained using eight algorithms, including MCSA, GWO, and WSO, are given in Figure 8a–h, while Figure 8i shows the trend of convergence during the iterative process. The optimal values of the stochastic parameters and their corresponding minimum energies are listed in Table 1. From the control disks that can be observed in the figures, it can be seen that the optimized parameters affect the control vertices, as well as the radius, and thus the direction and thickness of the curve. More obvious is the third DWB curve after the optimization of the WSO and other algorithms. The differences between the algorithms are found to be small in terms of the final shape, and the visual data in Table 1 shows that MCSA gained the smallest energy of the eight algorithms.

**Example** **2.** *This case uses CDWB curve to design a “snake” pattern, which consists of five DWB curves stitched together and satisfying G^2^ smooth continuum as a whole. An optimization model is developed with the objective of minimizing the energy of the curve, as expressed in Equation (33). According to the constraints, the optimization variables for this model are the parameters α_1_, α_2_, β_1_, and β_2_ for the jth (j = 2,3,4,5) DWB curve satisfying G^2^ geometric continuity condition.*

**Figure 9:**
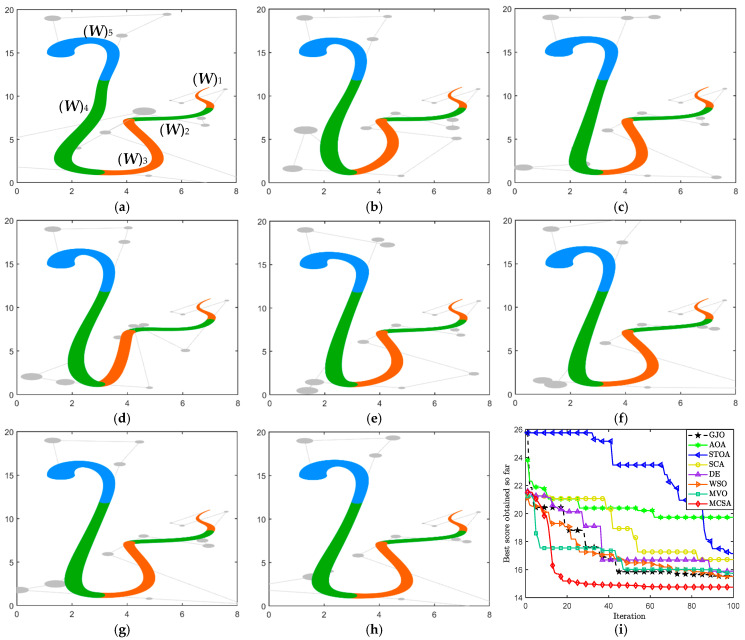
“Snake” pattern based on CDWB curve. (**a**) MVO, (**b**) AOA, (**c**) GJO. (**d**) STOA, (**e**) SCA, (**f**) DE. (**g**) WSO, (**h**) MCSA, (**i**) convergence curves.

The optimal parameter variables and the minimum energy obtained by the eight MAs are given in Table 2. Furthermore, the optimized results are visualized in Figure 9a–h, and Figure 9i shows the convergence curves. The results for MVO illustrate that the parameter values can significantly affect the effect of the curves and even lead to distortions in the overall shape. In combination with the energy data and the results, the curves obtained by MCSA and WSO with smaller energies are smoother at the nodes compared to the other algorithms. Based on the curve energy used to determine smoothness, MCSA achieves a minimum energy of 14.751305.

**Example** **3.** *This example is a “Chinese knot” pattern, designed by a CDWB curve, which consists of a total of eight DWB curves combined. In order to satisfy the overall G^1^ smooth continuity, the jth (j = 2, ..., 8) curve should satisfy Theorem 2. Given the DWB curve (**W**)_1_ and the part of the control disks of (**W**)_j_ (j = 2, ..., 8), an optimization model satisfying the smooth continuity of G^1^ in Section 3.1 is built with the curve energy as the objective function.*

**Figure 10:**
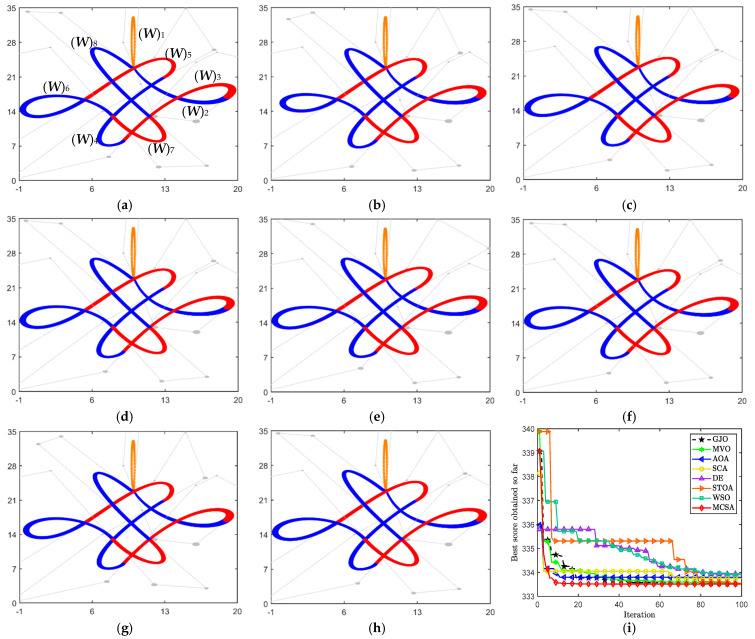
“Chinese knot” pattern based on CDWB curve. (**a**) SCA, (**b**) DE, (**c**) GJO. (**d**) MVO, (**e**) AOA, (**f**) STOA. (**g**) WSO, (**h**) MCSA, (**i**) convergence curves.

Table 3 lists the optimal values of the 14 variables and their corresponding minimum energies. Figure 10a–h show the modeling results obtained for the eight different algorithms. Furthermore, Figure 10i illustrates the convergence process in the iterations. Based on the optimal results and the convergence curves, MCSA shows a more competitive performance in the face of this example. It not only achieves the minimum curve energy, but also converges to the optimal value with a fast iteration rate.

### 3.5. Discussion

Section 3.4 exhibits the outcomes of numerical experiments, which cogently demonstrate that intelligent algorithms present highly efficacious and prominent approaches for resolving the shape optimization conundrums of CDWB curves. When considering G^1^ and G^2^ continuities of diverse complexity levels, a comprehensive numerical comparison among multiple algorithms, taking into account parameters such as convergence rate, convergence precision, minimum energy, and the smoothness of the CDWB curves, unequivocally validates that the introduced MCSA exhibits remarkable superiority. It not only showcases rapid convergence speed and high convergence precision, but also ensures a notably enhanced smoothness of the resultant disk Wang–Ball curves, thereby conclusively establishing their capacity to achieve an outstanding optimization effect.

## 4. Conclusions

This paper defines CDWB curves formed by the combination of *n* DWB curves and investigates the G^1^ and G^2^ continuity conditions at the splice nodes. Compared to conventional curves, disk curves remain constant during geometrical transformations such as rotations and have different widths through controlled radii, while a CDWB curve can satisfy more complex modeling designs than single curves. Based on the criterion of curve smoothness, we use the energy of the curve as the objective function, and constrain it with the G^1^ and G^2^ continuity conditions to build the optimization model, respectively. In addition, the competitive optimization algorithm MCSA is introduced to solve the previously established models. MCSA is an improved version of CSA, which not only inherits the strong search capability of CSA, but also improves the capabilities of exploiting and avoiding local optima with the combination of FO calculus, sinusoidal adjustment, and CCL strategy. Through comparison with a number of classical and novel algorithms, three examples numerically demonstrate that MCSA is superior in terms of finding solutions to CDWB curve shape optimization problems. This can also be applied in fields such as engineering design and manufacturing, computer-aided design, and animation production.

Although an optimization model using the energy method has been established, the impact of the weights of the center curve and radius function energies on the CDWB curve’s total energy and other performance indicators has not been fully studied, potentially leading to suboptimal results. Therefore, further investigating the impact of the weights of the two energy components on the total energy, how the energy weights affect other performance metrics of the curve, and more effective parameter adjustment strategies to better adapt to the shape optimization problems of CDWB curves with different levels of complexity will be the next research tasks.

## Figures and Tables

**Table 1:** Optimized results for Chinese character “弓” with G^1^ continuity.

Method	Parameters	Optimal Parameter Variables			Energy
		*j* = 2	*j* = 3	*j* = 4	
GWO	*α*_1_	3.496598	0.500000	0.500000	86.520164
	*α*_2_	0.170125	19.996942	1.119740	
MVO	*α*_1_	3.487348	0.500000	0.500000	86.528426
	*α*_2_	0.171274	17.372159	1.968351	
AOA	*α*_1_	3.533888	0.500000	0.500000	86.547738
	*α*_2_	0.272004	20.000000	0.636630	
SCA	*α*_1_	3.172702	0.500000	0.500000	86.585730
	*α*_2_	0.206337	19.371612	0.347748	
STOA	*α*_1_	3.484280	0.5	0.5	86.523149
	*α*_2_	0.162019	20	1.449695	
DE	*α*_1_	3.344544	0.501062	0.500148	86.558621
	*α*_2_	0.179524	15.355944	1.056753	
WSO	*α*_1_	2.921853	0.990801	0.504853	88.799267
	*α*_2_	3.801809	8.450565	2.576418	
MCSA	*α*_1_	3.502395	0.5	0.5	86.519192
	*α*_2_	0.171143	19.893545	0.583494	

**Table 2:** Optimized results for “Snake” pattern with G^2^ continuity.

Method	Parameters	Optimal Parameter Variables				Energy
		*j* = 2	*j* = 3	*j* = 4	*j* = 5	
MVO	*α*_1_	2.163511	0.571052	0.277113	1.601873	15.737175
	*α*_2_	4.916315	2.564152	3.260973	4.702275	
	*β*_1_	−2.446877	−2.949739	−1.829195	2.964973	
	*β*_2_	−0.958170	2.242326	−3.011651	−4.966703	
AOA	*α*_1_	1.843930	0.884693	0.932293	1.759613	19.728070
	*α*_2_	1.050236	5	3.014100	4.938082	
	*β*_1_	0.003230	2.4879 × 10^−9^	−0.001769	−0.001434	
	*β*_2_	0.000912	−0.001478	0.000915	−0.000947	
GJO	*α*_1_	2.106624	0.673132	0.734160	1.152351	15.532742
	*α*_2_	3.677985	2.439312	5	2.120650	
	*β*_1_	−0.005596	−5	−0.018117	1.689690	
	*β*_2_	−2.094051	−0.396819	−0.166906	1.782403	
STOA	*α*_1_	0.668488	1.255721	1.661235	1.445426	17.143764
	*α*_2_	4.179706	4.003217	4.989004	4.621070	
	*β*_1_	4.589853	3.480160	−1.228904	0.148168	
	*β*_2_	−3.804298	0.227467	−2.098405	2.097861	
SCA	*α*_1_	2.249720	0.717536	1.495918	1.359881	16.718694
	*α*_2_	5	2.814102	4.266499	1.688930	
	*β*_1_	0.034051	−4.179311	−3.063586	2.014430	
	*β*_2_	0.539825	−0.071023	4.231954	0.781479	
DE	*α*_1_	2.974248	0.602219	0.997978	1.465989	15.881302
	*α*_2_	1.861221	3.402069	3.447110	4.248911	
	*β*_1_	4.584556	−4.903025	−4.159690	−2.807080	
	*β*_2_	0.830291	3.095553	1.177691	0.287207	
WSO	*α*_1_	2.270619	0.628542	0.674676	1.874187	15.749756
	*α*_2_	1.162429	3.628808	3.926450	3.904971	
	*β*_1_	−0.265529	−4.413978	−2.020885	−4.086349	
	*β*_2_	1.983865	0.776000	−4.637694	1.392620	
MCSA	*α*_1_	2.773397	0.577761	0.484472	1.505580	14.751305
	*α*_2_	3.628415	2.362079	4.986970	1.668446	
	*β*_1_	3.687531	−4.998785	−2.516266	0.302868	
	*β*_2_	4.481423	−0.939683	2.034803	2.515662	

**Table 3:** Optimized results for the “Chinese knot” pattern with G^1^ continuity.

Method	Parameters	Optimal Parameter Variables							Energy
		*j* = 2	*j* = 3	*j* = 4	*j* = 5	*j* = 6	*j* = 7	*j* = 8	
SCA	*α*_1_	1.46618	0.75032	5	1.27254	0.5	1.36416	0.47092	333.74615
	*α*_2_	0.64864	3.96702	4.85923	5	5	1.88832	1.16434	
DE	*α*_1_	0.74481	1.17040	3.07958	1.45533	0.72109	1.27545	0.50809	333.90498
	*α*_2_	2.11867	4.54145	3.97695	1.80938	1.99623	3.09109	0.73686	
GJO	*α*_1_	0.5	1.05597	4.01045	1.33108	0.50665	1.25972	0.46537	333.51432
	*α*_2_	0.01622	5	4.72647	4.98566	3.46001	5	0.56538	
MVO	*α*_1_	0.5	1.06594	4.04700	1.29314	0.5	1.28461	0.46425	333.55311
	*α*_2_	0.18562	4.99617	3.41395	2.71411	3.54735	3.47489	4.68993	
AOA	*α*_1_	0.86792	0.90599	5	0.86462	0.5	1.25635	0.45000	333.79635
	*α*_2_	1.82486	2.88047	1.66113	3.60377	4.46127	4.10775	4.10775	
STOA	*α*_1_	0.57543	1.07952	3.91110	1.22169	0.50803	1.27179	0.47016	333.53220
	*α*_2_	0.01143	4.98317	4.16030	5	3.39809	3.90083	1.16007	
WSO	*α*_1_	1.23403	1.05216	4.40504	1.54213	0.55872	1.51185	0.51448	333.89544
	*α*_2_	1.83582	2.72107	1.74940	2.00836	3.47465	1.26459	1.17947	
MCSA	*α*_1_	0.500001	1.06141	4.10563	1.30932	0.50666	1.28952	0.46536	333.51118
	*α*_2_	2.05082	5.00000	4.99999	4.99997	4.31717	4.99939	4.81926	

## Data Availability

All data generated or analyzed during this study were included in this published article.

## Abbreviation

Abbreviation	Meaning
CDWB curves	Combined disk Wang–Ball (CDWB) curves
MCSA	Multi-strategy ameliorated chameleon swarm algorithm
FO calculus	Fractional-order calculus
CCL strategy	Crossover-based comprehensive learning strategy
SCA	Sine Cosine Algorithm
WSO	White Shark Optimizer
AOA	Arithmetic Optimization Algorithm
STOA	Sooty Tern Optimization Algorithm
GJO	Golden jackal optimization
DE	Differential evolutionary algorithms
GWO	Grey wolf optimizer
MVO	Multi-verse optimizer

## References

[1] Ball A.A. (1974). CONSURF, part 1: Introduction to the conic lofting title. Comput.-Aided Des..

[2] Ball A.A. (1975). CONSURF, part 2: Description of the algorithms. Comput.-Aided Des..

[3] Ball A.A. (1977). CONSURF, part 3: How the program is used. Comput.-Aided Des..

[4] Wang G.J. (1987). Ball curve of high degree and its geometric properties. Appl. Math. J. Chin. Univ..

[5] Said H.B. (1989). Generalized ball curve and its recursive algorithm. ACM Trans. Graph..

[6] Goodman T.N., Said H.B. (1991). Properties of generalized Ball curves and surfaces. Comput.-Aided Des..

[7] Goodman T.N., Said H.B. (1991). Shape preserving properties of the generalised Ball basis. Comput. Aided Geom. Des..

[8] Hu S.M., Wang G.Z., Jin T.G. (1996). Properties of two types of generalized ball curves. Comput.-Aided Des..

[9] Sederberg T.W., Farouki R.T. (1992). Approximated by interval Bézier curves. IEEE Comput. Graph. Appl..

[10] Tan J., Jiang P. (2006). Boundary and degree reduction of the interval Ball curves. J. Comput.-Aided Des. Comput. Graph..

[11] Chen F., Yang X., Yang W. (2002). Degree reduction of interval B-spline curves. J. Softw..

[12] Tuohy S.T., Maekawa T., Shen G., Patrikalakis N.M. (1997). Approximation of measured data with interval B-splines. Comput.-Aided Des..

[13] Chen F., Lou W. (2000). Degree reduction of interval Bézier curves. Comput.-Aided Des..

[14] Lin Q., Rokne J.G. (1998). Disk bézier curves. Comput. Aided Geom. Des..

[15] Chen F.L., Yang W. (2004). Degree reduction of disk Bézier curves. Comput. Aided Geom. Des..

[16] Chen X., Wang G. (2005). Disk Bézier approximation of equidistant curves. J. Softw..

[17] Seah H.S., Wu Z., Tian F., Xiao X., Xie B. Artistic brushstroke representation and animation with disk b-spline curve. Proceedings of the 2005 ACM SIGCHI International Conference on Advances in Computer Entertainment Technology.

[18] Hu G., Yang R., Wei G. (2023). Hybrid chameleon swarm algorithm with multi-strategy: A case study of degree reduction for disk Wang-Ball curves. Math. Comput. Simul..

[19] Terzopoulos D., Platt J., Barr A., Fleischer K. (1987). Elastically deformable models. Comput. Graph..

[20] Juhász I., Róth Á. (2019). Adjusting the energies of curves defined by control points. Comput.-Aided Des..

[21] Hu G., Li M., Wang X., Wei G., Chang C.-T. (2022). An enhanced manta ray foraging optimization algorithm for shape optimization of complex CCG-Ball curves. Knowl.-Based Syst..

[22] Hu G., Li M., Zhong J. (2022). Combined cubic generalized Ball surfaces: Construction and shape optimization using an enhanced JS algorithm. Adv. Eng. Softw..

[23] Rajeev S., Krishnamoorthy C.S. (1992). Discrete optimization of structures using genetic algorithms. J. Struct. Eng..

[24] Sowmya R., Premkumar M., Jangir P. (2024). Newton-Raphson-based optimizer: A new population-based metaheuristic algorithm for continuous optimization problems. Eng. Appl. Artif. Intell..

[25] Hu G., Du B., Wang X., Wei G. (2022). An enhanced black widow optimization algorithm for feature selection. Knowl.-Based Syst..

[26] Khatab M., El-Gamel M., Saleh A.I., El-Shenawy A., Rabie A.H. (2025). Coyote and Badger Optimization (CBO): A natural inspired meta-heuristic algorithm based on cooperative hunting. Commun. Nonlinear Sci. Numer. Simul..

[27] Tian Z., Gai M. (2024). Football team training algorithm: A novel sport-inspired meta-heuristic optimization algorithm for global optimization. Expert Syst. Appl..

[28] Hu G., Zhu X., Wang X., Wei G. (2022). Multi-strategy boosted marine predators algorithm for optimizing approximate developable surface. Knowl.-Based Syst..

[29] Chen D., Ge Y., Wan Y., Deng Y., Chen Y., Zou F. (2022). Poplar optimization algorithm: A new meta-heuristic optimization technique for numerical optimization and image segmentation. Expert Syst. Appl..

[30] Storn R., Price K. (1997). Differential evolution-a simple and efficient heuristic for global optimization over continuous spaces. J. Glob. Optim..

[31] Yu X., Jiang N., Wang X., Li M. (2023). A hybrid algorithm based on grey wolf optimizer and differential evolution for UAV path planning. Expert Syst. Appl..

[32] Zhao W., Wang L., Zhang Z. (2019). Atom search optimization and its application to solve a hydrogeologic parameter estimation problem. Knowl.-Based Syst..

[33] Hu G., Dou W., Wang X., Abbas M. (2022). An enhanced chimp optimization algorithm for optimal degree reduction of Said-Ball curves. Math. Comput. Simul..

[34] Braik M.S. (2021). Chameleon Swarm Algorithm: A bio-inspired optimizer for solving engineering design problems. Expert Syst. Appl..

[35] Wang J., Lv M., Li Z., Zeng B. (2023). Multivariate selection-combination short-term wind speed forecasting system based on convolution-recurrent network and multi-objective chameleon swarm algorithm. Expert Syst. Appl..

[36] Rajesh C., Sadam R., Kumar S. (2024). An evolutionary Chameleon Swarm Algorithm based network for 3D medical image segmentation. Expert Syst. Appl..

[37] Zhou J., Xu Z. (2023). Optimal sizing design and integrated cost-benefit assessment of stand-alone microgrid system with different energy storage employing chameleon swarm algorithm: A rural case in Northeast China. Renew. Energy.

[38] Dinh P.-H. (2023). Combining spectral total variation with dynamic threshold neural P systems for medical image fusion. Biomed. Signal Process. Control.

[39] Mostafa R.R., Ewees A.A., Ghoniem R.M., Abualigah L., Hashim F.A. (2022). Boosting chameleon swarm algorithm with consumption AEO operator for global optimization and feature selection. Knowl.-Based Syst..

[40] Rizk-Allah R.M., Hassanien A.E., Snášel V. (2022). A hybrid chameleon swarm algorithm with superiority of feasible solutions for optimal combined heat and power economic dispatch problem. Energy.

[41] Hu G., Yang R., Qin X., Wei G. (2023). MCSA: Multi-strategy boosted chameleon-inspired optimization algorithm for engineering applications. Comput. Meth. Appl. Mech. Eng..

[42] Mirjalili S. (2016). SCA: A Sine Cosine Algorithm for solving optimization problems. Knowl.-Based Syst..

[43] Braik M., Hammouri A., Atwan J., Al-Betar M.A., Awadallah M.A. (2022). White Shark Optimizer: A novel bio-inspired meta-heuristic algorithm for global optimization problems. Knowl.-Based Syst..

[44] Abualigah L., Diabat A., Mirjalili S., Elaziz M.A., Gandomi A.H. (2021). The Arithmetic Optimization Algorithm. Comput. Meth. Appl. Mech. Eng..

[45] Chopra N., Ansari M.M. (2022). Golden jackal optimization: A novel nature-inspired optimizer for engineering applications. Expert Syst. Appl..

[46] Dhiman G., Kaur A. (2019). STOA: A bio-inspired based optimization algorithm for industrial engineering problems. Eng. Appl. Artif. Intell..

[47] Mirjalili S., Mirjalili S.M., Lewis A. (2014). Grey Wolf Optimizer. Adv. Eng. Softw..

[48] Mirjalili S., Mirjalili S.M., Hatamlou A. (2016). Multi-Verse Optimizer: A nature-inspired algorithm for global optimization. Neural Comput. Appl..

